# Gene discovery in an invasive tephritid model pest species, the Mediterranean fruit fly, *Ceratitis capitata*

**DOI:** 10.1186/1471-2164-9-243

**Published:** 2008-05-23

**Authors:** Ludvik M Gomulski, George Dimopoulos, Zhiyong Xi, Marcelo B Soares, Maria F Bonaldo, Anna R Malacrida, Giuliano Gasperi

**Affiliations:** 1 Department of Animal Biology, University of Pavia, Piazza Botta 9, Pavia 27100, Italy; 2 Johns Hopkins Bloomberg School of Public Health, Baltimore, MD 21205-2179, USA; 3 Northwestern University's Feinberg School of Medicine, Chicago, IL 60614-3394, USA

## Abstract

**Background:**

The medfly, *Ceratitis capitata*, is a highly invasive agricultural pest that has become a model insect for the development of biological control programs. Despite research into the behavior and classical and population genetics of this organism, the quantity of sequence data available is limited. We have utilized an expressed sequence tag (EST) approach to obtain detailed information on transcriptome signatures that relate to a variety of physiological systems in the medfly; this information emphasizes on reproduction, sex determination, and chemosensory perception, since the study was based on normalized cDNA libraries from embryos and adult heads.

**Results:**

A total of 21,253 high-quality ESTs were obtained from the embryo and head libraries. Clustering analyses performed separately for each library resulted in 5201 embryo and 6684 head transcripts. Considering an estimated 19% overlap in the transcriptomes of the two libraries, they represent about 9614 unique transcripts involved in a wide range of biological processes and molecular functions. Of particular interest are the sequences that share homology with *Drosophila *genes involved in sex determination, olfaction, and reproductive behavior. The medfly *transformer2 *(*tra2*) homolog was identified among the embryonic sequences, and its genomic organization and expression were characterized.

**Conclusion:**

The sequences obtained in this study represent the first major dataset of expressed genes in a tephritid species of agricultural importance. This resource provides essential information to support the investigation of numerous questions regarding the biology of the medfly and other related species and also constitutes an invaluable tool for the annotation of complete genome sequences. Our study has revealed intriguing findings regarding the transcript regulation of *tra2 *and other sex determination genes, as well as insights into the comparative genomics of genes implicated in chemosensory reception and reproduction.

## Background

The medfly, *Ceratitis capitata*, is a highly invasive agricultural pest species that has expanded from its native range in sub-Saharan Africa to become a cosmopolitan species in less than 200 years. Its success as an invasive species is partially due to its unusually wide host range and its ability to adapt to a wide range of climatic conditions and habitats [1]. As such, it has become the target of extensive control programs and a model organism for the sterile insect technique (SIT), a method considered to be among the most efficient and environmentally friendly control procedures [2,3]. This technique, designed to reduce the size of the target population, is based on the release of sterile males that compete for wild females. Indeed, the medfly was the first non-drosophilid organism to be transformed [4], with the goal of introducing genes capable of improving genetic sexing systems for the SIT. Although molecular genetics studies of the medfly began in the early 1990s, at present (January 2008) only 182 putative coding sequences are known, almost half of which are fragmentary [5]. This lack of molecular data is in sharp contrast to the mass of data that has been accrued on the classical and population genetics of this model insect.

The number of published complete genome sequences has grown exponentially since the first two bacterial genomes were reported in 1995, with over 600 available as of 2008 [6]. These genome sequences include a number of important insect genomes, such as those of *Drosophila melanogaster*, the malarial mosquito, *Anopheles gambiae*, the silkworm *Bombyx mori*, and the honeybee *Apis mellifera *[7-10]. Numerous other insect genome-sequencing projects are in progress, including those for numerous species of *Drosophila*, mosquitoes of the genera *Aedes*, *Anopheles *and *Culex*, the cotton bollworm *Helicoverpa armigera*, the tobacco budworm *Heliothis virescens*, the human louse *Pediculus humanus*, the vector of Chagas disease *Rhodnius prolixus*, the tsetse fly *Glossina morsitans*, the sandfly *Lutzomyia longipalpis*, parasitic wasps of the genus *Nasonia*, the flour beetle *Tribolium castaneum*, and several aphids and ticks [6,11,12].

The initial goal of these genome sequence projects is to identify a complete set of genes and subsequently to determine their expression in different life stages and tissues and to characterize their regulation and function. Given that the haploid genome size of the medfly is relatively large (540 Mb), three times larger than that of *D. melanogaster*, the sequencing of the complete genome would be prohibitively expensive except by a large consortium.

To address the lack of sequence data available for the medfly, we have initiated a functional genomics approach based on expressed sequence tags (ESTs). ESTs represent a relatively quick and inexpensive technology for discovering new genes, for obtaining data on their expression and regulation, and for the construction of genome maps [13]. They are an ideal means for the rapid exploration of transcriptomes, especially those of species with large genome sizes. ESTs can also form a very solid basis for evolutionary studies.

The genetic information obtained from this EST initiative will be of enormous value for identifying and determining the functions of genes involved in a number of important biological processes, including sex determination, sex differentiation, reproduction, courtship behavior, and olfaction. Such processes represent ideal targets for the development of novel control methods and pest-monitoring systems. To target these biological processes we have utilised cDNA libraries derived from medfly embryos and adult heads as the source of our ESTs. The embryo library permits the identification of genes involved in sex determination and development whereas the head library permits the identification of genes involved in different behaviours, in olfaction etc. The availability of a large number of transcripts also permits the development of oligonucleotide-based microarrays that will facilitate the study of these biological processes by means of mass expression profile analyses.

Apart from its economic importance, the medfly also represents an alternative model dipteran species. *Drosophila melanogaster *is the model dipteran *par excellence*, but in many ways it is an atypical species. The availability of mosquito genomes has helped to balance this bias, and hopefully the medfly data presented here will also contribute to that end.

Here we present a comprehensive EST-based gene discovery project that has provided sequences of 11,885 transcripts and yielded novel insights into various biological activities of an important agricultural pest, the medfly.

## Results and Discussion

### Generation and assembly of medfly embryo and head ESTs

Two unidirectional, normalized cDNA libraries were constructed from embryos ranging in age from 30 min to 36 hr after oviposition and from adult male and female heads of flies ranging from 30 min to 8 days after emergence. Thus, the embryo library is representative of the transcriptome of embryos at different stages of development. The head library is representative of the transcriptome of adult heads of both sexes and different physiological states (immature, virgin, mated).

A total of 24,030 random cDNA clones from the two libraries were sequenced from the 5' end. These sequences, once trimmed of vector, contaminants, and low-quality sequences, yielded a total of 21,253 high-quality masked ESTs, with an average length of 700 bp for the embryo sequences and 723 bp for the head sequences, and representing over 15 megabases of medfly sequence.

The sequences from the two libraries were assembled separately using the Phrap program [14]: 7,173 of the embryo ESTs were assembled into 2,107 contiguous sequences (contigs), and the remaining 3,094 ESTs that were not redundant were classified as singlets. For the head ESTs, assembly resulted in 2,785 contigs (from 7087 ESTs) and 3,899 singlets. Contigs and singlets derived from the embryo ESTs are given the prefixes FC and FS, respectively, followed by a number. The head contigs and singlets have the prefixes HC and HS, respectively. The phrap program produces contigs consisting of a single-read which represent sequences that produced a match with other sequences but that could not be consistently assembled with these other reads. The highest number of ESTs in a single contig was 206 (HC2785), but very few contigs contained more than 10 ESTs. The distribution of the ESTs in contigs and singlets is illustrated in Table 1.

**Table 1:** EST assembly statistics

	Embryo Library	Head Library
Number of sequences	11512	12518
Number of high quality sequences	10267	10986
Number of putative transcripts (assembled sequences)	5201	6684
Number of contigs	2107	2785
Number of singlets	3094	3899
Number of contigs containing:		
1 EST	130	825
2–4 ESTs	1649	1734
5–10 ESTs	274	193
11–20 ESTs	38	23
21–40 ESTs	11	8
> 40 ESTs	5	2
Mean assembled sequence length (bp)	786	834

Almost 55% of the assembled embryo sequences and 29% of the assembled head sequences contained open reading frames (ORFs) with start codons that potentially encode at least 150 amino acids. However, given that ESTs are single-read sequences and that 5'-truncated cDNA inserts are not uncommon, we obtained a less stringent estimate of 69% for the embryo sequences and 36% for the head sequences when the presence or absence of the start codon was ignored (Table 1).

The sequences that lacked a putative ORF produced 43% hits in the case of the embryo library and only 19% hits for the head library. Of the assembled sequences containing a putative ORF, 89% of those derived from both the head and the embryo libraries had BLASTX matches in the non-redundant (nr) database. Subsequent TBLASTX analyses against the insecta set of EST sequences in the dbEST database increased this percentage to 91% in the embryo and 92% in the head. This finding suggests that perhaps 9% of the medfly transcripts, from the embryo or head, are highly divergent from their homologs in other organisms. It is probable that many of the sequences without putative ORFs and BLAST matches are non-coding sequences and may represent 5' or 3' UTRs.

Consistent with the expectation that the cDNA clones were sequenced from the 5' end, 98.4% of the assembled embryo sequences and 93.8% of the head sequences with hits in the nr database were encoded on the forward strand. The small proportion of assembled sequences that appeared to be encoded on the reverse strand may be the result of the cDNA being inserted in the opposite direction in the vector.

Almost 75% of the assembled embryo sequences and 44% of the assembled head sequences produced BLASTX hits against the nr database with an expectation, *e*, of less than 10^-5^. Well over 90% of the best hits were arthropod-derived sequences. Not surprisingly, of these arthropod sequences, 90% were *Drosophila *sequences, and of these, more than half pertained to *D. melanogaster *(Additional file 1, Table S1).

Only 58 of the best hits (18 for embryo and 40 for head sequences) were against *C. capitata *sequences, a finding that reflects the scarcity of medfly sequences in the databases (Additional file 2, Table S2). BLASTN analysis showed that three of the 13 sequences identified from a medfly male accessory gland cDNA library [GenBank:DQ406807, DQ406810, DQ406812] [15] were represented in the embryo (FC2089) and head assembled sequences (HC1979, HC2078, HC2666, HC2668). This finding has no bearing on the specificities of our libraries as all three genes (*Antigen-r5*, *Jafrac1 *and *virus-induced RNA 1*) are putatively involved in the immune pathway and in *Drosophila *are expressed in embryos and/or other adult tissues including the head.

Fifteen of the embryonic assembled sequences and 44 of the head sequences appeared to be of viral origin. Thirteen assembled sequences showed significant amino acid similarity to the polyproteins of the sacbrood [GenBank:AAT45735] and 16 to the Kakugo viruses [GenBank:NC_005876] previously identified in the honeybee. Another three sequences showed significant amino acid similarity to a virus polyprotein sequence isolated from *Varroa destructor *mites living on honeybee larvae. Twenty-three sequences showed significant amino acid identity to a cysteine-rich repetitive sequence in the U88 gene of the human herpesvirus 6 [GenBank:NC_001664] and another similarity to a highly repetitive sequence within the latency associated antigen gene of the ovine herpesvirus 2 [GenBank:AAL05844]. Single sequences showed similarity with the RNA-dependent RNA polymerase region of the 183-kDa protein of the Odontoglossum ringspot virus, the polymerase subunit of the influenza C virus, and the putative viral replicase of the prune dwarf virus. It is possible that some of these sequences represent retroviral elements.

Only 20 sequences appeared to have originated from the fly's bacterial flora, with homology to bacterial sequences of the genera *Bacteroides*, *Burkholderia*, *Escherichia*, *Haemophilus*, *Magnetococcus*, or *Staphylococcus*.

Sixty three of the transcripts showed significant homology to transposable elements; 11 of these were from the embryo library and the remaining 52 from the head library. The majority of these putative transposable elements belonged to the *mariner *(44) and *Tc1 *(15) families of transposable elements, but elements related to *hAT *family (two sequences, one to an element from *Danio rerio *and another to *hermit *from *Lucilia cuprina*), and to retrotransposons (two sequences related to the *D. melanogaster 1731 *retrotransposon) were also detected. The best hits for almost two-thirds of the *mariner*-like elements identified were previously identified elements from *C. capitata *or *Ceratitis rosa *(*e *values ranged from 1*e*-6 to 5*e*-25) [16].

### Annotation of the assembled sequences

The medfly ESTs were annotated with respect to *D. melanogaster*, which is not only the most extensively annotated genome but also relatively close to the medfly in evolutionary terms. Both species are members of the Acalyptratae and are estimated to have diverged from a common ancestor 80 – 100 million years ago [17,18]. Each medfly assembled sequence and singlet was assigned a gene ontology (GO) classification based the annotation of the best-hit *D. melanogaster *peptide obtained in BLASTX searches; thus, our annotations are at the "inferred from electronic annotation" (IEA) level of evidence. To avoid potential compounding of errors, *Drosophila *annotations assigned at the IEA level were not considered for the annotation of the medfly ESTs.

Of the 5,201 assembled embryo sequences, 74.5% (3876) produced best hits with an expectation, *e*, of <10^-5 ^against the *Drosophila *peptide database (containing 19,178 peptides), and 51.9% (2699) were assigned GO annotations. In the case of the head sequences, 39.6% (2,649 of 6,684) produced hits, and 31.1% (2,077) were assigned GO annotations.

The 5,201 embryo-derived and 6,684 head-derived assembled sequences presumably represent distinct transcripts. However, these numbers are likely to be an overestimate of the actual number of transcripts obtained, because ESTs derived from the same gene may not have been assembled into a single contig because of alternative splicing or sequence polymorphism. A total of 3,876 assembled embryo sequences produced best hits with 3,290 different *D. melanogaster *genes, suggesting a 15.1% redundancy in the assembled sequences. Extrapolating this redundancy value to the complete dataset, we estimate that the 5,201 assembled sequences represent about 4,400 genes expressed in the embryo. Likewise, for the head sequences, a total of 2,649 assembled medfly head sequences produced best hits with 2,304 different *D. melanogaster *genes, a 13% redundancy in the assembled sequences; thus, the 6,684 assembled sequences may represent about 5,815 genes expressed in the adult head.

Clearly, we can expect that there will be some overlap in the genes expressed in the sequences derived from the embryo and head library. To determine the extent of this overlap, the ESTs from the two libraries were pooled and reassembled using Phrap. This procedure generated a total of 9,614 assembled sequences (4,185 contigs and 5,429 singlets). Given that the two libraries when assembled separately gave rise to a total of 11,885 assembled sequences, we can estimate that approximately 2,271 sequences were shared between the two libraries, for an overlap of about 19%.

A summary of the allocation of the annotations to specific biological processes and molecular functions as classified by GO is presented in Additional files 3 and 4, Tables S3 and S4. A wide range of processes and functions are represented. Of particular interest in terms of the development of novel control methods for this pest species are the annotations related to sex determination, olfaction, and reproductive behavior.

### Genes involved in sex determination

In *Drosophila*, the primary sex determinant is the ratio of the number of X chromosomes to the number of sets of autosomes. When the ratio is 1 (XX:AA), the master switch gene, *Sex lethal *(*Sxl*), is activated and sets in motion a cascade of regulatory genes, *transformer (tra)*, *transformer-2 (tra2) *and *doublesex *(*dsx*), that result in female development. When the ratio is 0.5 (X:AA), *Sxl *is not activated, and male development proceeds. Although the medfly sex determination cascade is only partially characterized, it is clear that the initial levels differ from those of *Drosophila*. In the medfly, the primary sex determinant is a male-determining factor (M) on the Y chromosome. Thus, XX embryos develop into females and XY embryos into males. The medfly homolog of *tra*, *Cctra*, acts as the switch gene rather than the homologue of *Sxl*, *CcSxl*. The active product of the *Cctra *gene, CcTRA, which is present only in females, directs female-specific splicing of the *doublesex *(*dsx*) pre-mRNAs [19-21]. In this respect, the medfly sex determination pathway appears to have a greater affinity to that of *Musca domestica *than to that of *Drosophila *[22].

Of the three sex determination genes previously described in the medfly, *CcSxl*, *Ccdsx*, and *Cctra*, only *CcSxl *was identified among the medfly assembled sequences (Additional file 2, Table S2). However, 24 of the medfly assembled sequences shared homology with 13 *Drosophila *genes that have been implicated in sex determination (Table 2). Of particular interest was the sequence FC1744 from the embryo library, which shared 57%/73% amino acid identity/homology with the *transformer 2 *(*tra2*) sequence of *D. melanogaster*. FC1744 appears to be a full-length *tra2 *transcript. In *Drosophila tra2 *encodes a splicing regulator protein that contains an RNA recognition motif (RRM) flanked by two regions rich in arginine and serine residues (RS domains). The existence of a medfly *tra2 *homologue, *Cctra2*, has been hypothesized [20,21] but has not previously been described. It is thought that the CcTRA2 protein might interact with CcTRA to control both female-specific splicing of *Ccdsx *and the positive feedback loop established by the *Cctra *gene. The *Ccdsx *sequence contains conserved TRA/TRA2 binding sites close to the regulated splice site, suggesting that both TRA and TRA2 proteins are involved in the splicing process [20,21].

**Table 2:** Medfly assembled sequences with best-hit matches to *D. melanogaster *genes involved in sex determination

Medfly Sequence	*Drosophila *gene	Alignment Length (aa)	*e*-Value	Identity (%)	Similarity (%)
FC662	*groucho *(*gro*)	82	6E-07	29	50
FC1046	*sisterless A *(*sisA*)	180	3E-13	26	49
FC1310	*female lethal d *(*fl*(2)*d*)	493	3E-91	47	53
FC1664	*sans fille *(*snf*)	216	2E-99	84	88
FC1744	*transformer 2 *(*tra2*)	106	9E-32	57	73
FC2001	*intersex *(*ix*)	142	2E-54	71	85
FS1109	*hopscotch *(*hop*)	248	4E-50	39	63
FS1419	*deadpan *(*dpn*)	48	1E-10	75	83
FS1610	*Mes-4*	160	1E-62	65	80
FS1866	*groucho *(*gro*)	217	1E-126	97	98
FS2679	*Mes-4*	161	2E-42	40	54
FS2848	*Mes-4*	147	2E-19	36	51
HC1587	*modifier of mdg4 *(*mod(mdg4)*)	97	4E-34	60	80
HC2665	*lola like *(*lolal*)	127	9E-68	99	99
HS375	*sans fille *(*snf*)	80	1E-39	97	98
HS438	*modifier of mdg4 *(*mod(mdg4)*)	61	2E-16	55	70
HS653	*longitudinals lacking *(*lola*)	39	3E-18	100	100
HS900	*modifier of mdg4 *(*mod(mdg4)*)	263	7E-74	58	69
HS1176	*modifier of mdg4 *(*mod(mdg4)*)	73	3E-28	73	89
HS1648	CG3726	94	1E-31	71	79
HS2391	*modifier of mdg4 *(*mod(mdg4)*)	114	1E-37	57	78
HS2544	*longitudinals lacking *(*lola*)	247	1E-62	52	61
HS2947	*longitudinals lacking *(*lola*)	104	1E-50	83	89
HS3522	*modifier of mdg4 *(*mod(mdg4)*)	94	1E-17	46	67

**Figure 1:**
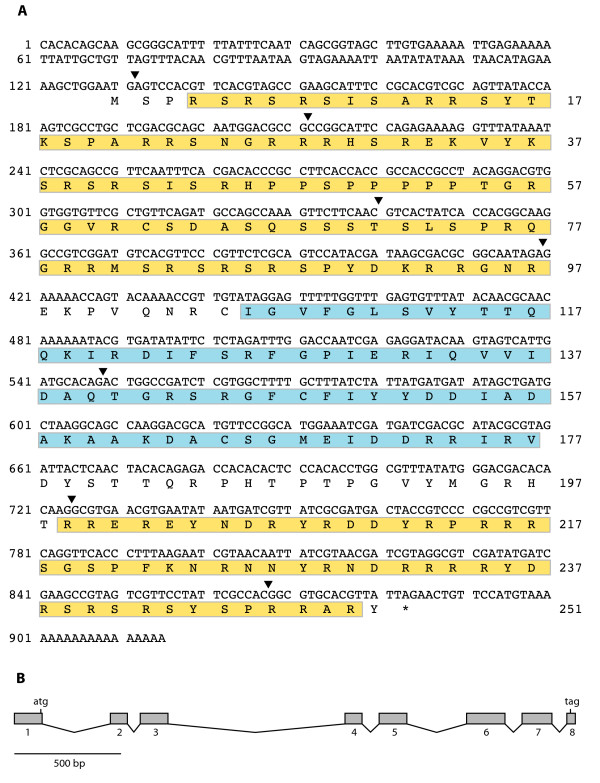
(**A**) Nucleotide and deduced amino acid sequences of the *Ceratitis capitata tra2 *gene (*Cctra2*) cDNA. The RNA recognition motif (RRM) is boxed in blue. The two arginine-rich/serine-rich regions (RS-domains) are boxed in yellow. The positions of the introns are indicated by triangles. (**B**) Genomic organization of the *Cctra2 *gene. The genomic sequence has been deposited in GenBank (accession no. EU437408).

The genomic sequence of the *Cctra2 *gene, amplified using a pair of primers designed in the 5' and 3' UTRs on the cDNA sequence of FC1744, is over 2.6 kb in length. Comparison of the genomic and cDNA sequences revealed the presence of eight exons (34 – 176 bp in length) and seven introns (64 – 834 bp in length). The splice sites all conformed to the GT-AG rule [23]. The positions of the introns were conserved with respect to the other tephritid *tra-2 *sequence from *Bactrocera oleae *[GenBank:AJ547623] and that of *M. domestica *[GenBank:AY847518]. The *tra2 *gene of *D. melanogaster *has seven exons rather than the eight present in *Cctra2*. This difference appears to be the result of the presence of an extra intron in *Cctra2 *within the *Drosophila *equivalent of exon 6. Furthermore, only two of the intron positions were conserved with respect to the *Drosophila tra2*. Figure 1 illustrates the cDNA sequence and the deduced 251 amino acid sequence of *Cctra2*. Amino acids 106 to 177 represent an RNA recognition motif (RRM) (*e *value, 7e-10) diagnostic of an RNA-binding protein [24]. The RRM is flanked by two arginine-rich/serine-rich regions (RS domains), which mediate protein-protein interactions to facilitate the formation of spliceosomal and regulatory splicing complexes [25]. Examination of the four EST sequences that comprise FC1744 revealed no indication of alternative splicing of the *Cctra2 *gene. RT-PCR analysis of different development stages/tissues (embryos, male and female larvae, adult heads and adult bodies) with primers located in the 5' UTR and exon 7 produced a single product of about 840 bp in each case, suggesting that the gene is not alternatively spliced (data not shown). The gene was expressed in both sexes and in all the life stages examined, although the transcripts present in the very early embryos may be of maternal origin. This expression pattern is very similar to that of *M. domestica *[22] but very different from that of *D. melanogaster *where at least five different *tra2 *transcripts are known, resulting from alternative promotors and differential splicing [26]. In *Drosophila*, the somatic transcripts are not sex-specific but two alternatively spliced transcripts are found only in the male germline [26].

**Figure 2:**
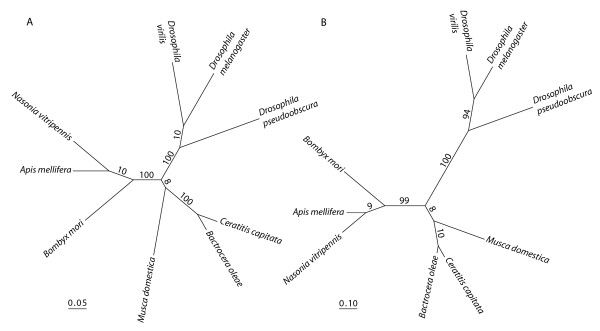
Phylogenetic analyses of the *tra2 *amino acid sequences from *C. capitata*, *B. oleae *(*Botra2*), *M. domestica*, *D. melanogaster*, *D. virilis*, *D. pseudoobscura*, *A. mellifera*, *N. vitripennis*, and *B. mori*. **A**. Neighbor-joining minimum evolution tree (ME-score = 1.750) with bootstrap values (percentage of 10,000 replications). The scale represents the mean character distance. **B**. Maximum-likelihood tree based on the Jones-Taylor-Thornton model of amino acid change (Ln Likelihood = -4070.80) with bootstrap values (percentage of 100 replications). The scale represents the expected number of amino acid substitutions per position.

The highest identity/similarity of the *Cctra2 *amino acid sequence was with the *tra2 *homologue from *B. oleae *(*Botra2*) ([GenBank:CAD67988]; 88%/93%). The phylogenetic relationships of the *tra2 *amino acid sequences from *C. capitata*, *B. oleae*, *M. domestica *[GenBank:AAW34233], *D. melanogaster *[GenBank:AAA62771], *D. virilis *[GenBank:AAB58114], *D. pseudoobscura *[GenBank:XP_001360605], *A. mellifera *[GenBank:XP_001121070], *Nasonia vitripennis *[GenBank:XP_001601106], and *Bombyx mori *[GenBank:AAX47001] are represented in the neighbor-joining tree (Figure 2). The sequences cluster according to the taxonomic relationships of the insect species. Thus, *Cctra2 *clusters with the other tephritid sequence *Botra2 *from *B. oleae*, and the two hymenopteran sequences, *Amtra2 *and *Nvtra2*, form a well-supported cluster, as do the three *Drosophila *sequences. In both trees, the *tra2 *products of the Tephritidae (Acalyptrate) appear to be more closely related to that of *M. domestica *(Calyptrate) than to those of the Drosophilidae (Acalyptrate). This topology is in agreement with those inferred from glucose-6-phosphate dehydrogenase [27], *white *[28], and alcohol dehydrogenase [29] and supports the evolutionary hypothesis in which the Tephritidae are closer to the Calyptrate Calliphoridae than to the Acalyptrate Drosophilidae [30]. The greater affinity of the medfly sex-determination system to that of the housefly than to that of *Drosophila *is further evidence of this evolutionary relationship [22].

Apart from their role in sex-determination, *tra2 *genes are also involved in male courtship behavior. The TRA2 protein interacts with TRA to regulate splicing of the *fruitless *gene (*fru*). Male-specific *fru *transcripts are essential for male courtship behavior [31,32].

Three of the other medfly assembled sequences that are putatively involved in sex determination share sequence homology with members of the three classes of primary X:A signal genes that encode transcription factors that regulate *Sxl *expression in *Drosophila*. The *sisterless A *gene belongs to the numerator class of primary signal genes and positively regulates *Sxl*, whereas *deadpan *is the only known denominator gene and negatively regulates *Sxl*. The third class of primary signal genes is represented by *groucho*, a maternal gene whose product is also a negative regulator of *Sxl*. The genes *female lethal(2)d *and *sans fille *are also involved in the autoregulation of *Sxl*. *Intersex *is required for the activity of DSXF, the female transcription factor product of *doublesex *[33]. In addition to their potential usefulness in comparative studies of the sex determination pathways, these genes and others expressed during embryogenesis may be useful for the development of genetic sexing strains and as targets for pest control programs.

### Genes involved in olfaction

The biological success, and hence the economic impact, of the medfly can be ascribed in part to the sensitivity and selectivity of its olfactory systems, which are essential for the location of plant hosts and for the detection of pheromones during the recognition and location of mates [34].

The olfactory signal transduction cascade in insects is facilitated by three main groups of molecules: odorant-binding proteins (OBPs), odorant receptors (ORs), and odorant-degrading enzymes (ODEs) [35]. A group of OBPs, the pheromone binding proteins (PBPs), are expressed in pheromone-responsive sensilla and bind to pheromone molecules [36].

OBPs are small, water-soluble proteins that are present in high concentration in olfactory and gustatory sensilla [37]. They are thought to solubilize hydrophobic odorant molecules and transport them through the hydrophilic environment in the hemolymph to the ORs on the cell surface. However, given the large number of OBPs present in many insect species, many of which display different odorant-binding specificities, it is probable that they play an active role in odorant recognition, perhaps acting as selective filters rather than as passive odorant shuttles [38,39]. Once the odorant/OBP complex has bound to the receptor, the OBP may be actively involved in terminating signal transmission by inactivating the odorant molecule [40].

Fifty-one potential OBP genes have been identified in *D. melanogaster *[38]. BLASTX analyses identified 29 medfly sequences with significant hits to 12 different *Drosophila *OBP genes (Table 3). All but two of these putative medfly OBP genes were derived from the head library. Fourteen of these putative OBPs that produced hits with the *Obp99c *gene also gave very significant hits with the previously identified medfly male-specific serum polypeptide (MSSP) family of genes [41,42]. These MSSP sequences are presumably members of the minus-C subfamily of OBPs, since they do not contain all six of the conserved cysteine residues that characterize insect OBPs [40]. The MMSPs, of which there are at least seven members classified into three subgroups, α, β and γ, appear to be non-olfactory OBPs, and it has been hypothesized that they may be involved in the binding and transportation of male specific sex pheromones [42].

**Table 3:** Medfly assembled sequences with best-hit matches to *D. melanogaster *odorant binding protein genes

	*Drosophila *Gene	Alignment Length (aa)	*e*-Value	Identity (%)	Similarity (%)
FS806	*Obp8a*	114	8E-15	33	52
FS1734	*Obp84a/Pbprp4*	73	1E-15	46	63
HC144	*Obp99c*	108	3E-14	38	51
HC245	*Obp99c*	108	4E-15	39	53
HC522	*Obp99c*	106	7E-17	38	53
HC725	*Obp56d*	114	5E-24	41	64
HC745	*Obp99c*	106	3E-14	40	53
HC984	*Obp99c*	112	8E-18	38	54
HC1012	*Obp56d*	114	5E-24	41	64
HC1070	*Obp99c*	100	8E-11	36	50
HC1099	*Obp99c*	104	8E-13	37	51
HC1147	*Obp99c*	104	8E-13	37	51
HC1321	*Obp56h*	126	1E-15	34	55
HC1480	*Obp99c*	108	7E-16	40	54
HC1570	*Obp28a/Pbprp5*	122	2E-27	45	59
HC1629	*Obp69a/Pbprp1*	126	4E-23	38	62
HC1947	*Obp19a*	124	1E-39	60	78
HC2050	*Obp99c*	105	1E-12	38	53
HC2054	*Obp44a*	124	1E-41	62	77
HC2068	*Obp99c*	114	8E-19	39	55
HC2265	*Obp19d/Pbprp2*	110	7E-23	42	64
HC2316	*Obp99c*	112	1E-17	38	54
HC2492	*Obp99c*	117	2E-15	39	51
HC2536	*Obp83a/Pbprp3*	157	4E-62	68	78
HS1079	*Obp19b*	149	5E-26	37	57
HS2127	*Obp99c*	107	2E-17	39	59
HS2225	*Obp84a/Pbprp4*	64	2E-15	53	71
HS2969	*Obp84a/Pbprp4*	112	9E-24	44	61
HS3757	*Obp19d/Pbprp2*	110	4E-12	32	55

Another putative OBP was identified during the BLASTX analyses against the nr database. The sequence HS1065, again a head-derived sequence, shares 71/88% amino acid identity/similarity (alignment length = 120aa, *e *= 2E-50) with the *An. gambiae *gene *Obp1 *[43].

ORs are a group of transmembrane proteins with very diverse sequences. The OBP/odorant complex interacts with the OR to initiate signal transmission from the outside of the neuron to the inside. Two putative medfly OR genes were identified in the head library (Table 4), one with a complete coding sequence with high amino acid identity to *Drosophila Or83b*. *Or83b*, unlike other OR genes, is highly conserved in other insects, and its presence is essential for olfaction. In fact, the *Or83b *homolog has already been isolated in the medfly [44] (Additional file 2, Table S2). The other putative medfly OR (HS336) identified in the head library shares homology with the *Drosophila Or59a *gene, which is expressed in the dorsal organ dome on the larval head, where it is involved in the detection of food odors, and particularly aromatic compounds containing a benzene ring [45-47]. *Or59a *appears not to be expressed in adult *Drosophila *olfactory organs [48] but is maximally expressed in the male accessory glands of adult *Drosophila *[49]. At least 60 putative OR genes have been identified in *D. melanogaster*, of which 43 are expressed in the antenna or maxillary palp [47]. In mosquitoes, 79 and 131 putative OR genes have been identified in *Anopheles gambiae *and *Aedes aegypti*, respectively [50,51]. Given the dramatic sequence divergence of the other ORs between different insect species, it is difficult to identify these sequences by sequence homology, which may explain why only two OR sequences were identified in our preliminary screening of the medfly sequences.

**Table 4:** Medfly assembled sequences with best-hit matches to *D. melanogaster *odorant receptor protein genes

	*Drosophila *Gene	Alignment Length (aa)	*e*-Value	Identity (%)	Similarity (%)
HS336	*Or59a*	68	2E-14	47	69
HS2079	*Or83b*	268	1E-136	91	94

Little is known about the genes involved in reception and behavior in the medfly. This gene discovery study thus represents a unique opportunity to explore the molecular bases of these behavioral traits in the reproductive biology of this important economic pest species. In the long term, the results of the study will aid the development of more efficient sex attractants for the detection, monitoring, and control of this species [52,53].

### Genes involved in reproductive behavior

A total of 27 assembled sequences shared homology with 20 *Drosophila *genes known to be involved in reproductive behavior (Table 5). In *Drosophila*, the majority of these genes are involved in male courtship behavior. Mutants for the gene *quick-to-court *initiate courtship toward virgin females abnormally quickly and also readily attempt to court other males [54]. The *prospero *gene, which is involved in nervous system development, can alter the age of onset of sexual behavior in males: Males carrying a single copy of a *prospero *mutation court and mate precociously [55]. Other mutations can result in little or no courtship behavior (*courtless *and *takeout*) and produce defects in spermatogenesis (*takeout*) [56,57]. Mutations in the *dunce *and *Calcium calmodulin kinase II *genes disrupt the ability of the male to learn to avoid courting males and mated females [58]. Males with a mutation at one of the clock genes, *timeless*, display extended copulation times [59] and those with the *lingerer *mutation court and copulate with females normally, but subsequently have great difficulty in disengaging their genitalia [60]. Hyper-excitability mutations in the potassium channels encoded by the *Shaker *gene result in courtship suppression. Other mutants such as *paralytic *and *slowpoke *affect the sodium channel and calcium activated potassium channel, respectively, and result in defective courtship song production [58]. Finally, mutations in a mitochondrial ribosomal protein gene, *technical knockout*, result in unsuccessful male courtship behavior, apparently because of a hearing impediment [61].

**Table 5:** Medfly assembled sequences with best-hit matches to *D. melanogaster *genes involved in reproductive behaviour

	*Drosophila *Gene	Alignment Length (aa)	*e*-value	Identity (%)	Similariy (%)	Gene Ontology
FC451	*technical knockout*	148	5E-60	77	82	male courtship behavior
FC774	*Esterase 6*	220	7E-74	58	75	sperm competition
FC1294	*lingerer*	261	1E-102	69	73	copulation
FC1371	*no on or off transient A*	76	7E-07	43	48	male courtship behavior, song production
FC1528	*courtless*	166	6E-91	95	98	male courtship behavior, spermatogenesis
FC2041	*no on or off transient A*	144	2E-82	79	89	male courtship behavior, song production
FS1306	*Dopa decarboxylase*	165	2E-82	84	94	courtship behavior
FS1728	*Fmr1*	76	8E-17	56	59	male courtship behavior
FS1871	*Shaker*	70	1E-06	45	50	courtship behavior
FS2108	*Calcium/calmodulin*	111	3E-59	97	99	courtship behavior-dependent protein kinase II
FS2293	*gomdanji*	67	2E-09	37	61	courtship behavior
FS2531	*Fmr1*	34	5E-12	97	97	male courtship behavior
FS2790	*quick-to-court*	259	9E-78	61	68	male courtship behavior
HC321	*takeout*	210	1E-47	41	62	circadian rhythm, feeding behavior, male courtship behavior, rhythmic behavior
HC1018	*courtless*	166	7E-91	95	98	courtship behavior, male meiosis, spermatogenesis
HC1681	*takeout*	245	4E-61	44	65	circadian rhythm, feeding behavior, male courtship behavior, rhythmic behavior
HC1876	*timeless*	260	6E-57	46	59	circadian behavior, copulation
HC2478	*dunce*	246	5E-84	67	69	circadian rhythm, courtship behavior, oogenesis, olfactory learning
HS48	*paralytic*	48	4E-09	60	66	male courtship behavior, song production, muscle contraction
HS355	*prospero*	100	1E-55	87	94	courtship behavior, sensory perception of taste
HS445	*slowpoke*	146	5E-77	98	100	male courtship behavior, song production, circadian rhythm
HS790	*quick-to-court*	95	5E-41	89	93	courtship behavior, male courtship behavior
HS1199	*ken and barbie*	288	6E-83	55	65	copulation, genitalia morphogenesis, insemination
HS2483	*takeout*	179	2E-32	39	59	circadian rhythm, feeding behavior, male courtship behavior, rhythmic behavior
HS2917	*lingerer*	35	3E-14	94	94	copulation
HS3081	*logjam*	238	6E-99	74	84	oviposition, protein carrier activity, intracellular protein transport
HS3537	*Sphingosine kinase 2*	178	1E-30	41	54	flight behavior, oviposition, signal transduction

One of the two medfly assembled sequences that may be involved in female reproductive behavior has homology to the *Drosophila logjam *(*loj*) gene. Females carrying mutations in *loj *mate normally and store sperm just as normal females do, but they do not lay eggs. The *loj *mutation has no observable effect on male courtship behavior and fertility. The gene encodes a member of a family of putative vesicle cargo receptor proteins that may mediate the transmission of positive signals for oviposition from the central and ventral nerve cord [62]. The other medfly sequence that may be involved in female reproductive behavior has homology to the *Sphingosine kinase 2 *gene. *Drosophila *females with a mutation in this gene have reduced flight activity and fecundity. The reduced fecundity of these *Sk2 *mutants is due to retention of mature eggs in the ovaries, which may be the result of compromised ovarian function or a defect in either sperm storage or the response to seminal fluid proteins [63].

The reproductive and sexual behavior of the medfly is relatively well studied [64-66]. Receptive females are attracted to aggregations (leks) of "signaling" males emitting a sex pheromone, which also acts as an attractant for other males. The male orientates towards the female, deflects his abdomen ventrally and begins to vibrate his wings in a continuous manner, apparently wafting a plume of pheromone from his everted rectal pheromone sac toward the female. After a while the male switches to a rhythmic backwards and forwards wing movement while continuing to vibrate rapidly. At this point the rectal pheromone sac is retracted, so the male does not appear to produce pheromone; the female, however, may be stimulated aurally by the sound of the wing movements and visually by rapid movements of the male's head. The male subsequently leaps onto the back of the female, buzzes his wings, and rocks his body back and forth before aligning himself to face the same direction as the female and attempting to copulate. Copulation usually lasts up to 3 hr. Throughout the courtship, the female can terminate the affair by merely leaving, dislodging the male, or by refusing to copulate. After insemination, the female's behavior changes from mate-searching to host-fruit location for oviposition [64].

The courtship behavior of *Drosophila *has been studied in far greater detail and involves a series of steps: orientation, following, tapping, wing vibration or "singing," and licking (of the female genitalia), followed by tail curling and copulation [58]. Although the courtship behavior of *Drosophila *differs from that of the medfly, it is probable that the underlying genetic bases of these behaviors are sufficiently similar to allow the genes identified to be used to modify or disrupt the medfly's reproductive behavior.

## Conclusion

The sequences obtained in this study represent the first major dataset of expressed genes in a tephritid species of agricultural importance. The availability of this resource will support the investigation of numerous questions regarding the biology of the medfly. EST libraries represent a rich source of polymorphic markers, be they SSRs or SNPs, that can be employed in high-throughput genotyping methods for population genetics and ecological studies [67]. The EST sequences will also be of utmost importance for any future project in which the genome of this organism is sequenced. In practical terms, the EST resource represents an arsenal of information that will allow us to develop new control tools, whether chemical or genetic, that are aimed at altering sex determination, reproductive traits and behavior, and host preference. The identification of these genes in *C. capitata *will also greatly facilitate the isolation of homologous genes in other tephritid species, as the medfly is by no means the only tephritid species of economic importance. It does, however, represent a model species for true fruit flies of the genera *Ceratitis*, *Bactrocera*, *Dacus*, *Anastrepha *and *Rhagoletis*, which include agricultural pests in several geographic areas worldwide. The medfly ESTs will also facilitate studies to elucidate the genetics underlying polyphagous and monophagous traits in pest and non-pest tephritid species. The sequences obtained in this study have been arrayed on a 22K microarray, which will make it possible for biologically important questions to be addressed by mass expression profile analyses.

## Methods

### Flies

An established strain, ISPRA, was chosen for the creation of the cDNA libraries. ISPRA was established in 1968 at the European Community Joint Research Centre, Ispra, Italy, with wild medflies from Sicily and Greece. The strain has been maintained in the quarantine facility at the University of Pavia, Italy since 1979. Standard larval and rearing methods were used [68]. For the embryo library, two separate collections of eggs at <30 min to 36 hr after oviposition were carried out, with each collection offset by 9 hr (i.e., in the early morning and afternoon). The eggs were filtered from the water and rinsed with distilled water, then with 0.02% Triton X-100, and finally with diethylpyrocarbonate (DEPC) treated water. To obtain adults for the head library, a standard laboratory rearing cage was set up with about 600 less than 1 day old adults. Twelve males and 12 females were removed from the cage and used for RNA extraction at intervals of 24 hr for 8 days.

### cDNA library construction

For the embryo library, total RNA was extracted from approximately 1 g (wet weight) of eggs from each collection using Trizol (Invitrogen) according to the manufacturer's instructions, followed by treatment with DNase (DNAfree, Ambion). An equal quantity of total RNA from the two extractions was pooled prior to poly(A)^+ ^RNA purification. For the head library, total RNA was immediately extracted separately from the male and female heads from each collection using Trizol, followed by treatment with DNase. An equal quantity of total RNA from the male head and female head extractions was pooled prior to poly(A)^+ ^RNA purification.

First-strand cDNA synthesis was primed with an oligo(dT) containing a *Not*I restriction site. The double-stranded cDNA was ligated to an *EcoR*I adaptor, digested with *Not*I, and cloned directionally into a *Not*I- and *Eco*RI-digested pT7T3-Pac phagemid vector [69]. The cDNA inserts were flanked by a library-specific 3' linker tag sequence (5'-*Not*I-TAAGGTCGAG-3' in the embryo library and 5'-*Not*I- TCGACACAAT-3' in the head library) and 5' linker (5'-*EcoR*I-GGCACGAGG-3'). Both libraries were normalized [69].

### Sequencing and contig assembly

Randomly selected clones were sequenced from the 5' end using the M13 reverse sequencing primer (5'-AGCGGATAACAATTTCACACAGGA-3') with an Applied Biosystems 3730 DNA analyzer. Base-calling and low quality sequence trimming were achieved using Phred [70], and vector sequences were trimmed using Cross-match [71]. Repeat sequences were masked using RepeatMasker [72]. The sequences were assembled using Phrap [14]. The resulting assembled sequences were used to perform BLAST searches locally on a Macintosh G5 Unix workstation and on locally installed sequence databases, including the non-redundant protein sequence database and the *Drosophila*, *Anopheles gambiae*, and *Apis mellifera *protein databases. BLAST searches were performed using the low-complexity filter with the low-complexity sequences masked. A similarity was considered significant if the *e *value was lower than 10^-5^. GO annotations were derived from the best-hit *Drosophila *sequences and were obtained for each assembled sequence using FlyBase [73]. The presence of putative ORFs in the assembled sequences (with and without the start codon) was determined using Flip 2.0.2, with the minimum length set to 150 amino acids [74]. The sequences reported in this study have been deposited in GenBank under accession numbers [GenBank: FG068301 – FG089553].

### PCR-based cloning of *Cctra2*

Two primers based on the sequence of FC1744, Tra2-26f (5'- tcaatcagcggtagcttgtg-3') and Tra2-939r (5'-acgtgtgtttgtttgtttgct-3'), were used to amplify the sequence of the putative *Cctra2 *gene from genomic DNA isolated from the ISPRA strain. Amplification was performed using the AccuPrime Taq DNA Polymerase High Fidelity Kit (Invitrogen Srl, Milan) using the following conditions: an initial denaturing step at 94°C for 1 min, followed by 30 cycles of 30 sec at 94°C, 30 sec at 56°C, and 3 min 30 sec at 68°C, with a final extension of 10 min. Amplification products were cloned using the TOPO TA cloning kit (Invitrogen) and sequenced on both strands using the Big Dye Ready Reaction kit on an ABI 310 DNA Genetic Analyzer (Applied Biosystems, Foster City, CA).

### RT-PCR-based transcript detection

For transcript detection by RT-PCR, total RNA was extracted using Trizol (according to the manufacturer's instructions; Invitrogen, Milan, Italy) from pools of ~250 embryos in age ranges of 0–5, 5–10, 10–15, 15–20, 20–25 and 25–30 hr after oviposition; individual third instar larvae; and pools of eight heads and two headless bodies of 1- and 4-day-old adult virgin male and female flies. DNA was extracted from the same samples using the Trizol DNA extraction protocol. The larvae were sexed using a PCR technique [75]. cDNA was synthesized from 2.5 μg of RNA using the Cloned AMV First-Strand cDNA Synthesis Kit (Invitrogen, Milan, Italy). The primers used for the RT-PCR were Tra2-26f and Tra2-901r (5'-gcgaataggaacgactacgg-3'). The medfly glucose-6-phosphate dehydrogenase [GenBank: S67872] housekeeping gene was amplified as a control using the primers G6PDH-196f (5'-ttgtcatctttggtgcttcg-3') and G6PDH-372r (5'-ccggttgcaccttcatgtat-3'). To control for genomic DNA contamination, RT-PCR was also performed on samples in which cDNA synthesis had been performed in the absence of reverse transcriptase. RT-PCR was performed using 5% of the synthesized cDNA with the following cycle conditions: 94°C for 2 min, 30 cycles at 94°C for 30 sec, 56°C for 30 sec, 72°C for 1 min, and a final extension at 72°C for 10 min. The amplification products were electrophoresed on 1.5% or 2% agarose gels.

### Phylogenetic analysis

Multiple alignments of putative amino acid sequences were performed using the PRALINE server with the standard progressive strategy [76], and neighbor-joining minimum evolution trees were obtained using PAUP 4.0b10 [77]. Maximum-likelihood trees were obtained using the Jones-Taylor-Thornton model of amino acid change in Phylip version 3.67 [78].

## Authors' contributions

LMG, GD, ARM and GG conceived the study, and participated in its design and coordination. LMG performed RNA isolation, genomic sequencing and RT-PCR analyses. MBS and MFB prepared the libraries and performed cDNA sequencing. LMG and ZX performed sequence processing, assembly, annotation and bioinformatic analyses; LMG, GD, ARM and GG drafted the manuscript. All authors read and approved the final manuscript.

## Supplementary Material

Additional file 1 Table S1. Distribution of BLASTX best hits against the non-redundant protein database (nr). Click here for file

Additional file 2 Table S2. Assembled sequences with best-hit matches to known *C. capitata *sequences. Click here for file

Additional file 3 Table S3. Gene Ontology classification: Molecular Function. Click here for file

Additional file 4 Table S4. Gene Ontology classification: Biological Process. Click here for file
