# Supplementary material for: Gene discovery in an invasive tephritid model pest species, the Mediterranean fruit fly, Ceratitis capitata
Source: BMC Genomics. 2008 May 23;9:243. doi: 10.1186/1471-2164-9-243 (PMC2427042; doi:10.1186/1471-2164-9-243)
Supplement: Additional file 1 — Table S1. Distribution of BLASTX best hits against the non-redundant protein database (nr). [file 1471-2164-9-243-S1.doc]

**Table S1** Taxonomic distribution of BLASTX best hits against the non-redundant protein database (nr)

|  | Embryo Library | |  | Head Library | |
| --- | --- | --- | --- | --- | --- |
| Phylum | Hits | % Hits |  | Hits | % Hits |
|  |  |  |  |  |  |
| Arthropoda | 3776 | 97.29 |  | 2719 | 92.58 |
| Chordata | 52 | 1.34 |  | 99 | 3.37 |
| Echinodermata | 8 | 0.21 |  | 4 | 0.14 |
| Nematoda | 8 | 0.21 |  | 3 | 0.10 |
| Platyhelminthes | 1 | 0.03 |  | 4 | 0.14 |
| Protozoa | 8 | 0.21 |  | 29 | 0.99 |
| Viruses | 15 | 0.39 |  | 44 | 1.50 |
| Fungi | 6 | 0.15 |  | 10 | 0.34 |
| Bacteria | 5 | 0.13 |  | 16 | 0.54 |
| Plants | 1 | 0.03 |  | 3 | 0.10 |
| Synthetic | 1 | 0.03 |  | 6 | 0.20 |
