# Supplementary material for: Gene discovery in an invasive tephritid model pest species, the Mediterranean fruit fly, Ceratitis capitata
Source: BMC Genomics. 2008 May 23;9:243. doi: 10.1186/1471-2164-9-243 (PMC2427042; doi:10.1186/1471-2164-9-243)
Supplement: Additional file 2 — Table S2. Assembled sequences with best-hit matches to known C. capitata sequences. [file 1471-2164-9-243-S2.doc]

**Table S2.** Assembled sequences with best-hit matches to *C. capitata* sequences

|  | *Ceratitis* Gene | Alignment  Length (aa) | *e*-Value | Identity  (%) | Similarity  (%) |
| --- | --- | --- | --- | --- | --- |
|  |  |  |  |  |  |
| FC970 | *lethal of scute* | 126 | 3E-46 | 75 | 76 |
| FC1136 | *heat shock protein 83* | 195 | 6E-86 | 84 | 84 |
| FC1362 | *ribonuclease* | 95 | 5E-48 | 100 | 100 |
| FC1504 | *heat shock-like protein* | 296 | 1E-142 | 88 | 88 |
| FC1543 | *sex-lethal* homolog CcSXL | 289 | 1E-140 | 86 | 86 |
| FC1554 | *Superoxide dismutase* (Cu-Zn) | 142 | 7E-79 | 100 | 100 |
| FC1836 | *mapotgé* protein | 191 | 1E-105 | 98 | 100 |
| FC1972 | *heat shock protein 83* | 213 | 1E-116 | 99 | 100 |
| FC2032 | *mapotgé* protein | 298 | 1E-170 | 99 | 99 |
| FC2046 | ribosomal protein P2 | 113 | 2E-22 | 54 | 54 |
| FC2060 | 20S proteasome *α*5 subunit | 226 | 1E-119 | 97 | 98 |
| FC2061 | 60S acidic ribosomal protein P0 | 317 | 1E-154 | 89 | 89 |
| FS474 | *mariner* transposase | 27 | 4E-06 | 96 | 96 |
| FS477 | *unknown* | 113 | 3E-37 | 73 | 78 |
| FS1221 | *heat shock protein 83* | 198 | 1E-102 | 93 | 93 |
| FS1384 | *Cu/Zn superoxide dismutase* | 131 | 1E-72 | 98 | 98 |
| FS2511 | *6-phosogluconate dehydrogenase* | 115 | 2E-59 | 100 | 100 |
| FS2716 | *alcohol dehydrogenase 2* | 212 | 1E-116 | 99 | 99 |
| HC144 | *male-specific protein* | 124 | 2E-66 | 97 | 98 |
| HC172 | *NADH dehydrogenase* subunit 4 | 42 | 2E-15 | 92 | 95 |
| HC245 | *male specific serum polypeptide α1* | 144 | 6E-68 | 87 | 88 |
| HC268 | *Ccmar2* transposase | 86 | 5E-08 | 39 | 54 |
| HC278 | *mariner* transposase | 59 | 9E-25 | 86 | 93 |
| HC288 | *Vitellogenin-1* precursor | 71 | 2E-34 | 95 | 97 |
| HC464 | *mariner* transposase | 50 | 7E-22 | 96 | 98 |
| HC522 | *male specific serum polypeptide γ1* | 127 | 6E-52 | 78 | 79 |
| HC745 | *male specific serum polypeptide α1* | 122 | 8E-65 | 98 | 98 |
| HC871 | *mariner* transposase | 41 | 5E-11 | 87 | 92 |
| HC984 | *male specific serum polypeptide γ1* | 142 | 3E-61 | 81 | 82 |
| HC1022 | *Superoxide dismutase* (Cu-Zn) | 143 | 4E-79 | 100 | 100 |
| HC1070 | *male specific serum polypeptide β3* | 121 | 3E-62 | 96 | 96 |
| HC1099 | *male specific serum polypeptide β3* | 143 | 6E-67 | 88 | 88 |
| HC1147 | *male specific serum polypeptide β3* | 143 | 6E-67 | 88 | 88 |
| HC1480 | *male specific serum polypeptide α1* | 144 | 1E-68 | 88 | 88 |
| HC1515 | *Vitellogenin-1* precursor | 268 | 1E-136 | 92 | 93 |
| HC1743 | *cytochrome c oxidase* subunit 1 | 63 | 2E-25 | 92 | 96 |
| HC1751 | 20S proteasome α5 subunit | 241 | 1E-131 | 99 | 99 |
| HC1847 | *cytochrome b* | 100 | 1E-27 | 69 | 69 |
| HC2050 | *male specific serum polypeptide β3* | 144 | 8E-65 | 86 | 86 |
| HC2068 | *male specific serum polypeptide γ1* | 129 | 2E-63 | 91 | 92 |
| HC2239 | *Vitellogenin-2* precursor | 395 | 0 | 91 | 91 |
| HC2271 | *heat shock protein 83* | 172 | 3E-84 | 92 | 92 |

**Table S2** (continued) Medfly assembled sequences with best hit matches to *C. capitata* sequences

|  | *Ceratitis* Gene | Alignment  Length (aa) | *e*-Value | Identity  (%) | Similarity  (%) |
| --- | --- | --- | --- | --- | --- |
|  |  |  |  |  |  |
| HC2287 | *20S proteasome α5 subunit* | 226 | 1E-119 | 97 | 98 |
| HC2316 | *male specific serum polypeptide γ1* | 142 | 8E-62 | 82 | 83 |
| HC2479 | *ribosomal protein P2* | 47 | 3E-17 | 100 | 100 |
| HC2492 | *male-specific protein* | 144 | 1E-67 | 87 | 88 |
| HC2574 | *ribonuclease* | 95 | 8E-48 | 100 | 100 |
| HC2584 | *heat shock protein 83* | 219 | 1E-118 | 100 | 100 |
| HC2617 | *NADH dehydrogenase* subunit 4 | 53 | 4E-19 | 86 | 92 |
| HC2703 | *alcohol dehydrogenase 2* | 250 | 1E-140 | 100 | 100 |
| HS356 | *gag*-like protein | 31 | 1E-07 | 83 | 96 |
| HS571 | 60S acidic ribosomal protein P0 | 195 | 1E-104 | 99 | 99 |
| HS716 | *gag*-like protein | 238 | 2E-72 | 57 | 71 |
| HS2079 | odorant receptor *Or83b* | 259 | 1E-145 | 99 | 99 |
| HS2090 | *heat shock protein 83* | 234 | 1E-122 | 91 | 91 |
| HS2127 | *male specific serum polypeptide γ1* | 117 | 2E-32 | 55 | 71 |
| HS2156 | *gag*-like protein | 200 | 5E-61 | 55 | 76 |
| HS2301 | *env*-like protein | 152 | 8E-26 | 35 | 63 |
