# Supplementary material for: Gene discovery in an invasive tephritid model pest species, the Mediterranean fruit fly, Ceratitis capitata
Source: BMC Genomics. 2008 May 23;9:243. doi: 10.1186/1471-2164-9-243 (PMC2427042; doi:10.1186/1471-2164-9-243)
Supplement: Additional file 3 — Table S3. Gene Ontology classification: Molecular Function. [file 1471-2164-9-243-S3.doc]

**Table S3** Distribution of the ESTs in Molecular Function

| Gene Ontology Term | Medfly Embryo  Sequences | Medfly Head  Sequences | *Drosophila* Genes [79] |
| --- | --- | --- | --- |
|  |  |  |  |
| All molecular functions | 2274 | 1798 | 8852 |
| antioxidant activity | 8 | 16 | 34 |
| binding | 754 | 843 | 2474 |
| antigen binding | 1 | 0 | 1 |
| carbohydrate binding | 1 | 9 | 48 |
| cofactor binding | 1 | 4 | 10 |
| drug binding | 7 | 11 | 22 |
| hormone binding | 2 | 2 | 9 |
| isoprenoid binding | 2 | 8 | 9 |
| lipid binding | 19 | 31 | 72 |
| ion binding | 37 | 185 | 106 |
| neurotransmitter binding | 1 | 5 | 52 |
| nucleic acid binding | 406 | 338 | 982 |
| nucleotide binding | 61 | 111 | 102 |
| odorant binding | 2 | 15 | 53 |
| peptide binding | 7 | 6 | 59 |
| protein binding | 244 | 284 | 929 |
| steroid binding | 3 | 5 | 8 |
| catalytic activity | 1066 | 1006 | 3302 |
| helicase activity | 52 | 18 | 106 |
| hydrolase activity | 427 | 463 | 1538 |
| isomerase activity | 33 | 36 | 77 |
| kinase activity | 137 | 109 | 371 |
| ligase activity | 71 | 89 | 182 |
| lyase activity | 43 | 46 | 125 |
| oxidoreductase activity | 147 | 195 | 530 |
| transferase activity | 334 | 244 | 852 |
| chaperone regulator activity | 1 | 0 | 1 |
| enzyme regulator activity | 106 | 113 | 269 |
| enzyme activator activity | 29 | 28 | 69 |
| enzyme inhibitor activity | 19 | 40 | 87 |
| GTPase regulator activity | 52 | 40 | 91 |
| kinase regulator activity | 23 | 20 | 49 |
| phosphatase regulator activity | 14 | 7 | 30 |
| motor activity | 21 | 27 | 72 |
| microtubule motor activity | 17 | 6 | 37 |
| signal transducer activity | 86 | 162 | 515 |
| receptor binding | 16 | 35 | 135 |
| morphogen activity | 1 | 0 | 7 |
| receptor activity | 45 | 65 | 423 |
| receptor signaling protein activity | 23 | 61 | 64 |
| structural molecule activity | 135 | 185 | 405 |
| transcription regulator activity | 249 | 122 | 609 |
| transporter activity | 200 | 261 | 650 |
