# Supplementary material for: Gene discovery in an invasive tephritid model pest species, the Mediterranean fruit fly, Ceratitis capitata
Source: BMC Genomics. 2008 May 23;9:243. doi: 10.1186/1471-2164-9-243 (PMC2427042; doi:10.1186/1471-2164-9-243)
Supplement: Additional file 4 — Table S4. Gene Ontology classification: Biological Process. [file 1471-2164-9-243-S4.doc]

**Table S4** Distribution of the ESTs in Biological Process

| Gene Ontology Term | Medfly Embryo  Sequences | Medfly Head  Sequences | *Drosophila* Genes [79] |
| --- | --- | --- | --- |
|  |  |  |  |
| All biological processes | 1793 | 1953 | 7393 |
| cellular processes | 840 | 982 | 4958 |
| cell communication | 231 | 396 | 863 |
| developmental process | 553 | 452 | 2244 |
| cell differentiation | 115 | 83 | 1058 |
| cellularization | 19 | 13 | 89 |
| aging | 15 | 9 | 63 |
| death | 84 | 92 | 186 |
| embryonic development | 119 | 99 | 531 |
| pattern specification | 107 | 76 | 411 |
| pigmentation during development | 9 | 22 | 65 |
| post-embryonic development | 1 | 2 | 657 |
| regulation of gene expression, epigenetic | 22 | 16 | 145 |
| sex determination | 12 | 12 | 27 |
| sex differentiation | 3 | 9 | 57 |
| stem cell maintenance | 1 | 8 | 18 |
| growth | 12 | 13 | 130 |
| regulation of growth | 12 | 10 | 77 |
| metabolic process | 1185 | 1393 | 3142 |
| biological regulation | 58 | 72 | 1455 |
| regulation of enzyme activity | 2 | 7 | 55 |
| reproduction | 198 | 133 | 698 |
| reproductive behavior | 13 | 14 | 87 |
| response to stimulus | 161 | 256 | 975 |
| behavior | 75 | 66 | 452 |
| response to stress | 99 | 88 | 211 |
| sensory perception | 11 | 47 | 248 |
